# Absolute GABA spectroscopy with MEGA-PRESS and watermapping in sensorimotor and visual cortex and correlation to handedness

**DOI:** 10.1186/2047-783X-19-S1-S28

**Published:** 2014-06-19

**Authors:** Georg Oeltzschner, Nienke Hoogenboom, Thomas Baumgarten, Hans-Jörg Wittsack, Alfons Schnitzler

**Affiliations:** 1 Institute of Clinical Neuroscience and Medical Psychology, Heinrich Heine University, 40225, Düsseldorf, Germany; 2 Department of Diagnostic and Interventional Radiology, Heinrich Heine University, 40225, Düsseldorf, Germany

## Background

Quantification of γ-aminobutyric acid (GABA) concentrations in the brain has been the subject of many studies [1]. Nevertheless, quantification procedures vary substantially, mainly in terms of choice of the concentration reference. While in some cases creatine (Cr) or *N*-acetyl aspartate (NAA) are assumed to be of quasi-constant concentration (intra and inter subject), this may not be the case in pathologies, so that the use of an unsuppressed water reference should prove more reliable. In this work, a “water concentration ground truth” from the MRS voxel is pulled from a watermapping technique presented in [2].

## Methods

All measurements are carried out on a clinical 3T whole-body scanner (Siemens MAGNETOM Trio A Tim System, Siemens Healthcare AG, Erlangen) with a 12-channel head matrix coil. MEGA-PRESS [3] spectra are acquired from the desired voxel (TR = 1500 ms, TE = 68 ms, V = 27 mL). This includes water-suppressed (NEX = 128) and non-suppressed (NEX = 8) spectra for the water reference. After that, the five required imaging sequences for watermapping are recorded, followed by a high-res 3D MPRAGE scan for segmentation purposes. Spectroscopy data are processed with jMRUI v4 (http://www.mrui.uab.es/mrui/). Processing includes zero fitting (1024 data points), apodising (Gauß, 5 Hz), zero-order phasing of the NAA peak to 180° and HLSVD-filtering of residual water and lipid contamination. The GABA peak at 3 ppm is fitted with the AMARES routine, employing a Gaussian single peak with relative phase of 0°. The unsuppressed water signal is phase corrected manually and also quantified with AMARES. Water maps are calculated from the five single measurements with the freely available software predictMS [2]. The high-res 3D MPRAGE dataset is segmented by the SPMv8 (http://www.fil.ion.ucl.ac.uk/spm/) routine into GM, WM and CSF parts which are coregistered onto the water map. The MRS voxel information are read from the raw data (Siemens RDA) with a MATLAB (The Mathworks Inc., Natick/MA) routine and transformed into a binary mask. Thus, the absolute water content and the fractions of GM, WM and CSF in the voxel can be calculated from the water and tissue maps. Calculation of the absolute GABA concentration is carried out with T_2,GM_ = 100 ms, T_2,WM_ = 70 ms, T_1,CSF_ = 4160 ms, T_2,CSF_ = 500 ms, T_1,GABA_ = 800 ms and T_2,GABA_ = 130 ms. The method is applied to 16 healthy volunteers (8M, 8F, Æ age 30.5 ± 10.8 y) who underwent a handedness dominance test (HDT) by Steingrüber previously. MRS voxels were placed in the left (LMOT) and right (RMOT) sensorimotor cortex area (centered on the respective „hand knob“) as well as in the occipital visual cortex area. Furthermore, a measure for the asymmetry of GABA concentrations over LMOT and RMOT is introduced as ASYM = (LMOT-RMOT) / (LMOT+RMOT).

## Results

Table 1 shows the absolute GABA concentration values and volume fractions of the different tissue types. GABA estimates for LMOT and RMOT are almost identical, while the VIS value is significantly higher (*p* <*0.05* for both). This has previously been shown in [4]. Segmentation values are in agreement with literature [5]. Absolute GABA estimations are notably higher than typical literature values [5]. This is probably due to the fact that standard concentration estimation as in [5] assumes tissue specific mean values for the water content. These standard values are notably lower than the actual individual water content values that are acquired in this work (not shown).

**Figure 1:**
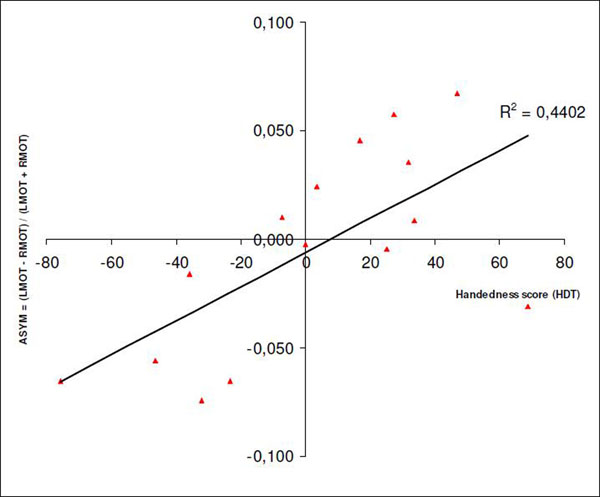
GABA laterality across left and right sensorimotor cortex is correlated with handedness.

Fig. 1 shows the laterality in LMOT and RMOT GABA as a function of the HDT handedness score (-100 = purely left-handed, +100 = purely right-handed). Preliminary data of 16 healthy controls suggest that a strong hand preference is associated with a strong GABA laterality in the primary motor cortex. The primary motor area responsible for the dominant hand shows higher GABA estimates, which is in line with recent findings (“more GABA, better performance”, [6]).

**Table 1:** Tissue composition for grey matter (GM), white matter (WM) and cerebrospinal fluid (CSF) and GABA concentration estimates for all three ROIs LMOT, RMOT (left/right primary motor cortex) and VIS (visual cortex).

	fraction of voxel volume / %			concentration [mM]
	
	GM	WM	CSF	GABA
**LMOT**	31 ± 4	57 ± 6	11 ± 4	**1.74 ± 0.34**

**RMOT**	29 ± 3	59 ± 9	10 ± 6	**1.75 ± 0.29**

**VIS**	58 ± 2	30 ± 4	11 ± 3	**1.95 ± 0.38**

## Conclusions

This work features an extended spectroscopy method for the exact quantitative estimation of GABA concentrations in sensorimotor and visual cortex. Estimation of the absolute concentration reference was performed via a watermapping technique. Resulting mean GABA concentrations were 1.74 ± 0.34 mM (LMOT), 1.75 ± 0.29 mM (RMOT) and 1.95 ± 0.38 mM (VIS). These estimates are notably higher than estimates acquired with standard quantification routines. In 16 healthy controls, a laterality of the GABA distributions between left and right primary motor cortex was found. Data suggests that the degree of GABA laterality is linked to the degree of handedness of the controls.

This work was supported by the Sonderforschungsbereich SFB 974, TP 7.
